# Differential Glial Activation in Early Epileptogenesis—Insights From Cell-Specific Analysis of DNA Methylation and Gene Expression in the Contralateral Hippocampus

**DOI:** 10.3389/fneur.2020.573575

**Published:** 2020-11-16

**Authors:** Toni C. Berger, Magnus D. Vigeland, Hanne S. Hjorthaug, Cecilie G. Nome, Erik Taubøll, Kaja K. Selmer, Kjell Heuser

**Affiliations:** ^1^Department of Neurology, Oslo University Hospital, Oslo, Norway; ^2^University of Oslo, Oslo, Norway; ^3^Department of Medical Genetics, Oslo University Hospital and University of Oslo, Oslo, Norway; ^4^Division of Clinical Neuroscience, Department of Research and Innovation, Oslo University Hospital, Oslo, Norway; ^5^National Centre for Epilepsy, Oslo University Hospital, Sandvika, Norway

**Keywords:** epilepsy, NeuN, TLE, glia, neuron, gene expression, DNA methylation, epigenetics

## Abstract

**Background and Aims:** Morphological changes in mesial temporal lobe epilepsy with hippocampal sclerosis (mTLE-HS) are well-characterized. Yet, it remains elusive whether these are a consequence of seizures or originate from a hitherto unknown underlying pathology. We recently published data on changes in gene expression and DNA methylation in the ipsilateral hippocampus (ILH) using the intracortical kainate mouse model of mTLE-HS. In order to explore the effects of epileptic activity alone and also to further disentangle what triggers morphological alterations, we investigated glial and neuronal changes in gene expression and DNA methylation in the contralateral hippocampus (CLH).

**Methods:** The intracortical kainic acid mouse model of mTLE-HS was used to elicit status epilepticus. Hippocampi contralateral to the injection site from eight kainate-injected and eight sham mice were extracted and shock frozen at 24 h post-injection. Glial and neuronal nuclei were sorted by flow cytometry. Alterations in gene expression and DNA methylation were assessed using reduced representation bisulfite sequencing and RNA sequencing. The R package edgeR was used for statistical analysis.

**Results:** The CLH featured substantial, mostly cell-specific changes in both gene expression and DNA methylation in glia and neurons. While changes in gene expression overlapped to a great degree between CLH and ILH, alterations in DNA methylation did not. In the CLH, we found a significantly lower number of glial genes up- and downregulated compared to previous results from the ILH. Furthermore, several genes and pathways potentially involved in anti-epileptogenic effects were upregulated in the CLH. By comparing gene expression data from the CLH to previous results from the ILH (featuring hippocampal sclerosis), we derive potential upstream targets for epileptogenesis, including glial *Cox2* and *Cxcl10*.

**Conclusion:** Despite the absence of morphological changes, the CLH displays substantial changes in gene expression and DNA methylation. We find that gene expression changes related to potential anti-epileptogenic effects seem to dominate compared to the pro-epileptogenic effects in the CLH and speculate whether this imbalance contributes to prevent morphological alterations like neuronal death and reactive gliosis.

## Introduction

Epileptogenesis describes the transformation of a normally functioning brain into an epileptic brain (1, 2). For mesial temporal lobe epilepsy with hippocampal sclerosis (mTLE-HS), this process often involves an initial incident (i.e., prolonged febrile seizure, inflammation, or cerebral trauma), followed by a clinically silent latent phase, and, ultimately, seizures of increasing frequency and severity (3). Pathological hallmarks of mTLE-HS are well-characterized in both humans and in animal models and predominantly consist of progressive neuronal cell death and reactive gliosis (4–14). The underlying mechanisms of these features remain elusive, and their further disentanglement is of paramount importance for the development of truly anti-epileptogenic drugs (15, 16).

In this paper, we use a combined analysis of cell-specific gene expression and DNA methylation to investigate epileptogenesis in a mouse model for mTLE-HS. Gene expression by means of RNA sequencing is a well-established approach for investigating biological function (17, 18). A cell-specific approach, i.e., the separation of neurons and glia prior to downstream analysis, has been used in various previous studies (19–21) and facilitates the detection of more subtle effects and the determination of the cellular origin of the observed DNA methylation and gene expression alterations (22).

DNA methylation contributes to cell-specific gene expression (23–26) and is altered in both epileptic human tissue (27) and animal models of epilepsy (22, 28–30). Amendable by, among other things, neuronal activity (31), nutrition (32), and newer epigenetic tools (33), it represents a modifiable potential upstream mechanism in epileptogenesis.

**Figure 1:**
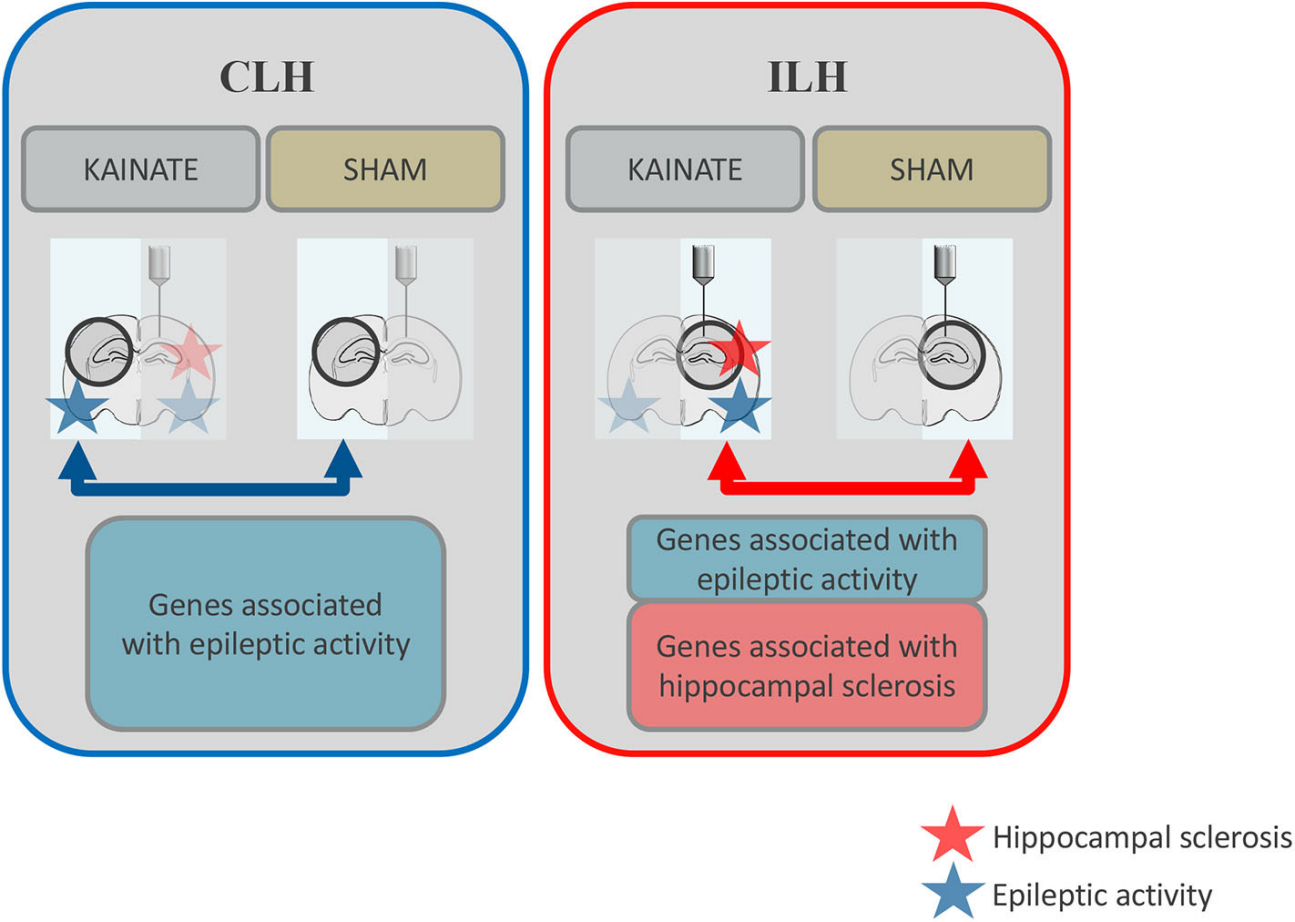
Schematic overview: Differential gene expression in the contralateral and ipsilateral hippocampi at 24 h after status epilepticus induction. Both the ipsilateral and contralateral hippocampi were exposed to epileptic activity (upon a status epilepticus lasting several hours), but only the ipsilateral hippocampus gradually develops morphological alterations such as reactive gliosis and neuronal death (hippocampal sclerosis). In this study, gene expression was compared between epileptic mice (kainate injected over the right, ipsilateral, hippocampus) and sham mice (saline injected at the same location) for the contralateral hippocampus (CLH, *blue arrow*). This data was compared to previous data on gene expression from the ipsilateral hippocampus (ILH, *red arrow*). Differentially expressed genes associated with epileptic activity are represented by the *blue boxes*. Differentially expressed genes potentially associated with morphological alterations typical of mesial temporal lobe epilepsy with hippocampal sclerosis (mTLE-HS) are represented by the *red box*.

We recently published a study on neuronal and glial DNA methylation and gene expression changes at 24 h post-kainate-induced status epilepticus, a time point relevant to early epileptogenesis (11, 34). These findings from the ipsilateral hippocampus (ILH) revealed a number of significant gene expression alterations in both neurons and glia. We further found a number of epilepsy-relevant genomic loci with a significant association of differential gene expression and differential DNA methylation (22). These observations originated from the intracortical kainate mouse model, where both hippocampi are exposed to epileptic activity but only the ILH gradually develops morphological changes (e.g., neuronal death and reactive gliosis) similar to human mTLE-HS (11) (Figure 1). In contrast, the contralateral hippocampus (CLH) is only exposed to epileptic activity and regarded as “free from morphological alterations” (11, 35). As such, it is often used as an internal control for the ILH (11).

The aims of this study were to on glia- and neuron-mediated downstream effects of epileptic activity based on gene expression changes in the CLH. We further elaborated on potential upstream targets for hippocampal sclerosis and epileptogenesis within genes exclusively differentially expressed in the ILH (and not CLH). Lastly, we explored the potential role of DNA methylation on cell-specific gene expression in early epileptogenesis.

## Methods

The methods applied in this study follow the same protocol as previously described in Berger et al. (22). Raw data are available under GEO accession GSE153976.

### Animals

Adult male C57/BL6N mice (Janvier Labs, France), acquired at an age of 8 weeks, were acclimatized for 4 weeks in a controlled environment (21–23°C, 12-h dark/light cycles). One to four animals were housed per cage, with water and food available *ad libitum*. All animal procedures were approved by the Norwegian Food Safety Authority (project number FOTS: 14198), the Center for Comparative Medicine, Oslo University Hospital and the University of Oslo.

### Intracortical Kainic Acid Mouse Model of Mesial Temporal Lobe Epilepsy With Hippocampal Sclerosis

We used the intracortical mouse model for mTLE-HS, described in detail by Bedner et al. (11), to elicit status epilepticus. In brief, the mice were anesthetized with a mixture of medetomidine (0.3 mg/kg, i.p.) and ketamine (40 mg/kg, i.p.) and kept on a heating blanket. For mice in the kainic acid group, kainate (70 nl, 20 mM, Tocris) was injected above the right hippocampus (= ipsilateral) by a Hamilton pipette (Hamilton Company, NV) at a depth of 1.7 mm at anteroposterior −2 mm, lateral +1.5 mm in relation to bregma. After the procedure, anesthesia was stopped with atipamezole (300 mg/kg, i.p.). Buprenorphine (0.1 mg/kg, s.c.) was applied at 4 and 12 h after the intervention. In order to ensure successful execution of technical procedures, only animals displaying convulsive seizures (Racine grade 5) within the first 4 h after termination of the procedures were included in further analysis. For sham animals, 0.9% NaCl was used instead of kainate for the intracortical injection.

### Tissue Collection and Pooling

Cervical dislocation was performed 24 h after status epilepticus, and hippocampi were extracted. Thereafter, each hemisphere was placed in a 2-ml polypropylene tube, instantly shock frozen in liquid nitrogen, and stored at −80°C. Left hippocampi (= contralateral) were pooled in 2-ml tubes from four (kainic acid group, *n* = 4; sham group, *n* = 4) or two (kainic acid group, *n* = 4; sham group, *n* = 4) mice prior to further processing. The number of mice amounted to eight per group (eight kainic acid and eight sham) and the number of biological samples to three per group (three samples in the kainic acid and three samples in the sham group). Tissue was kept on dry ice during pooling.

### Fluorescence-Activated Nuclear Sorting

Cell nuclei were sorted into NeuN+ nuclei (referred to as neurons) and NeuN– nuclei (referred to as glia) by a modified version of Jiang et al. (36) (for technical limitations and restrictions in interpretability, see *Limitations*). Hippocampi were placed on ice immediately after pooling, and 1 ml homogenization buffer was added. GentleMACS dissociator (Miltenyi) was used to homogenize the tissue. The homogenate was subsequently filtered through a 70-μm filter and debris removed by density gradient centrifugation using Debris Removal Solution (Miltenyi). Nuclear pellets were resuspended in 100 μl incubation buffer per 1 × 10^6^ nuclei and Anti-NeuN Alexa Fluor 488 (Merck Millipore) added (0.1 μg/ml per sample). The samples were incubated for 1 h on ice, protected from light. Adult mouse liver was used as a NeuN-negative control sample and processed in parallel with hippocampal tissue. The nuclei were sorted into NeuN+ and NeuN– fractions using a FACSAria (BD Biosciences), followed by centrifugation, and pellets were resuspended in lysis buffer for DNA and RNA isolation. For further details, see Supplementary Document.

### Isolation of DNA and Total RNA From Sorted Nuclei

MasterPure Complete DNA and RNA Purification Kit (Epicenter) was used to extract DNA from sorted nuclei. DNA purity was evaluated on NanoDrop and the DNA concentration assessed on Qubit (DNA HS assay). Total RNA was extracted using the mirVana miRNA Isolation Kit (Ambion) and RNA was up-concentrated with the RNA Clean & Concentrator-5 kit (Zymo Research). RNA integrity and concentration were analyzed on Bioanalyzer with the RNA Pico Kit (Agilent Technologies). For further details, see Supplementary Document.

### Reduced Representation Bisulfite Sequencing

A modified version of the gel-free protocol provided by Boyle et al. (37) was used for reduced representation bisulfite sequencing (RRBS) library preparation. Main changes comprised the inclusion of a two-sided size selection before bisulfite conversion and sample pooling after completion of single libraries. Libraries representing the contralateral and ipsilateral hemispheres were prepared and sequenced in parallel, and sequencing pools contained either 14 libraries run twice on NextSeq500 (50% PhiX spike-in, 75-bp single reads) or15 libraries sequenced over two lanes on HiSeq2500 (10% PhiX spike-in, 50-bp single reads). The library preparation procedure is described in detail in Supplementary Document.

### High-Throughput mRNA Sequencing

SMART-Seqv4 Ultra Low InputRNA Kit for Sequencing (Takara Bio) was used to amplify messenger RNA (mRNA) from total RNA, and the resulting complementary DNA (cDNA) was used as the input in library preparation with the ThruPlex DNAseq Kit (Rubicon Genomics). Libraries representing the contralateral and ipsilateral hemispheres were prepared and sequenced in parallel, and sequencing pools contained either 12 libraries sequenced on NextSeq500 (75-bp single reads) or 27 libraries sequenced over three lanes on HiSeq3000 (150-bp paired-end reads). Details regarding mRNA sequencing (mRNAseq) library preparation are given in Supplementary Document.

### Computational Methods

#### Bioinformatic Handling and Quality Control of mRNAseq Data

The mRNAseq reads were trimmed with Trim Galore! v0.4.3 and aligned by the Rsubread (the R interface of the Subread software) (38). Quality control of the BAM files was done with Picard/CollectRnaSeqMetrics. The featureCounts function of Rsubread was used for counting the number of reads mapping uniquely to each gene, based on the comprehensive gene annotation for mm10 in the GENCODE release M16 (www.gencodegenes.org/mouse/release_M16.html). Only reads aligning to mRNA regions were used in further analysis.

The expression levels (normalized counts) of a neuronal gene (*Rbfox3*), glial genes (*Aldh1l1, Cx3cr1*, and *Mbp*), as well as pericyte (*Pdgfrb*) and endothelial (*Pecam1*) genes were visualized to verify the enrichment of neurons and glia in the NeuN+ and NeuN– fractions. In order to validate our cell sorting procedures and discover outliers, a multidimensional scaling plot of the mRNAseq data was produced. For this, we used the edgeR function plotMDS to compute point coordinates, using the top 100 most variable genes, and ggplot2 (39) to produce the final plots.

#### Bioinformatic Handling and Quality Control of RRBS Data

The RRBS raw data underwent trimming with Trim Galore! v0.4.3, with parameters “–rrbs–illumina,” and quality control with FastQC. Alignment was done with Bismark v0.20 (powered by Bowtie2) using the mouse genome mm 10 as reference. The Picard tool CollectRrbsMetrics v2.18.15 was used for quality control of the BAM files.

An MDS plot of the RRBS data set was produced in a similar fashion to the mRNAseq, using the 100 most variable loci.

The bisulfite conversion rates were estimated in two ways. Firstly, by Picard/CollectRrbsMetrics, which measures the conversion of non-CpG cytosines. This statistic may be unreliable in neurons, where the methylation of non-CpG cytosines occurs with non-negligible frequency. To account for this, we also performed an alternative estimate of the conversion rates directly from the untrimmed fastq files, targeting the (unmethylated) cytosines added in the end-repair step of the RRBS preparation (private bash script). Samples whose conversion rate estimates were below 98% in both methods were excluded.

#### Annotation

Coordinates of the genes, exons, and introns were obtained from the M16 release of GENCODE's comprehensive annotation, restricted to autosomal genes. Annotation of CpG sites was performed with the R package annotatr (40), supplying details of the gene regions overlapping each CpG. In particular, promoter regions were defined as the 1-kb segments upstream of the transcription start sites, and upstream regions were defined as ranging from −5 to −1 kb relative to the transcription start sites.

#### Analysis of Differential Gene Expression

Analysis of differential gene expression between the kainic acid group and the sham group samples was performed with the R package edgeR (41). Preparatory steps included removal of genes without the official HGNC symbol, removal of genes with a low read count (determined by the edgeR function filterByExpr with default parameters), and normalization adjusting for different library sizes (done with calcNormFactors). The differential gene expression analysis followed a standard edgeR workflow based on a quasi-likelihood negative binomial generalized log-linear model fitted to the count data. Data from glial and neuronal cells were analyzed separately. The significance threshold was set to a false discovery rate (FDR) of 25%.

#### Analysis of Differential DNA Methylation

Loci exhibiting differential DNA methylation between the kainic acid group and the sham group samples were identified with edgeR, following a workflow for RRBS data recently published by the edgeR authors (42). In brief, this treats the methylated and unmethylated counts at each locus as independent variables following a negative binomial distribution. As for differential gene expression, the differential DNA methylation analysis was carried out separately for neuronal and glial cells, with a FDR of 25% as the significance threshold. Preparatory steps included removing all CpG sites where more than 10% of the samples had either very low coverage (< 8 reads) or very high coverage (>99.5 quantile across all sites and samples). In addition to the a standard differential DNA methylation analysis of individual CpG sites, aggregated analyses were performed for various genomic regions defined by the gene annotation, including upstream segments, promoters, UTR5's, exons, introns, gene bodies (i.e., the union of all exons and introns of a specific gene), and UTR3's. For the aggregated analysis, the input was the mean counts across all the covered CpGs within the region.

#### Combined Differential Gene Expression and Differential DNA Methylation Analysis

In order to identify genes for which both gene expression and DNA methylation differed significantly between the kainic acid group and sham group, a combined analysis of differential gene expression and (aggregated = differentially methylated regions) differential DNA methylation was performed for each genomic feature. For each feature type (upstream, promoter, UTR5, exon, intron, gene body, and UTR3), only the genes surviving filters in the corresponding aggregated differential DNA methylation data set were kept and used as inputs in a new differential gene expression analysis. Co-incidence of differential gene expression and differential DNA methylation was declared for features surviving a FDR cutoff of 25% in both analyses.

#### Functional Enrichment Analysis

Enrichment analyses of Gene Ontology (GO) and Kyoto Encyclopedia of Genes and Genomes (KEGG) pathways were performed with the goana and kegga functions of edgeR, with the parameter species = “Mm”.

### Selection of Relevant Gene Ontology and Kyoto Encyclopedia of Genes and Genomes Terms

Epileptogenesis-relevant GO and KEGG terms in neurons and glia were selected manually among the complete lists of respective terms in Supplementary Table (sheets 2, 3, 5, 6, 22, 23, 25, and 26) based on reviews on the subject (4, 15) and personal knowledge.

## Results

**Figure 2:**
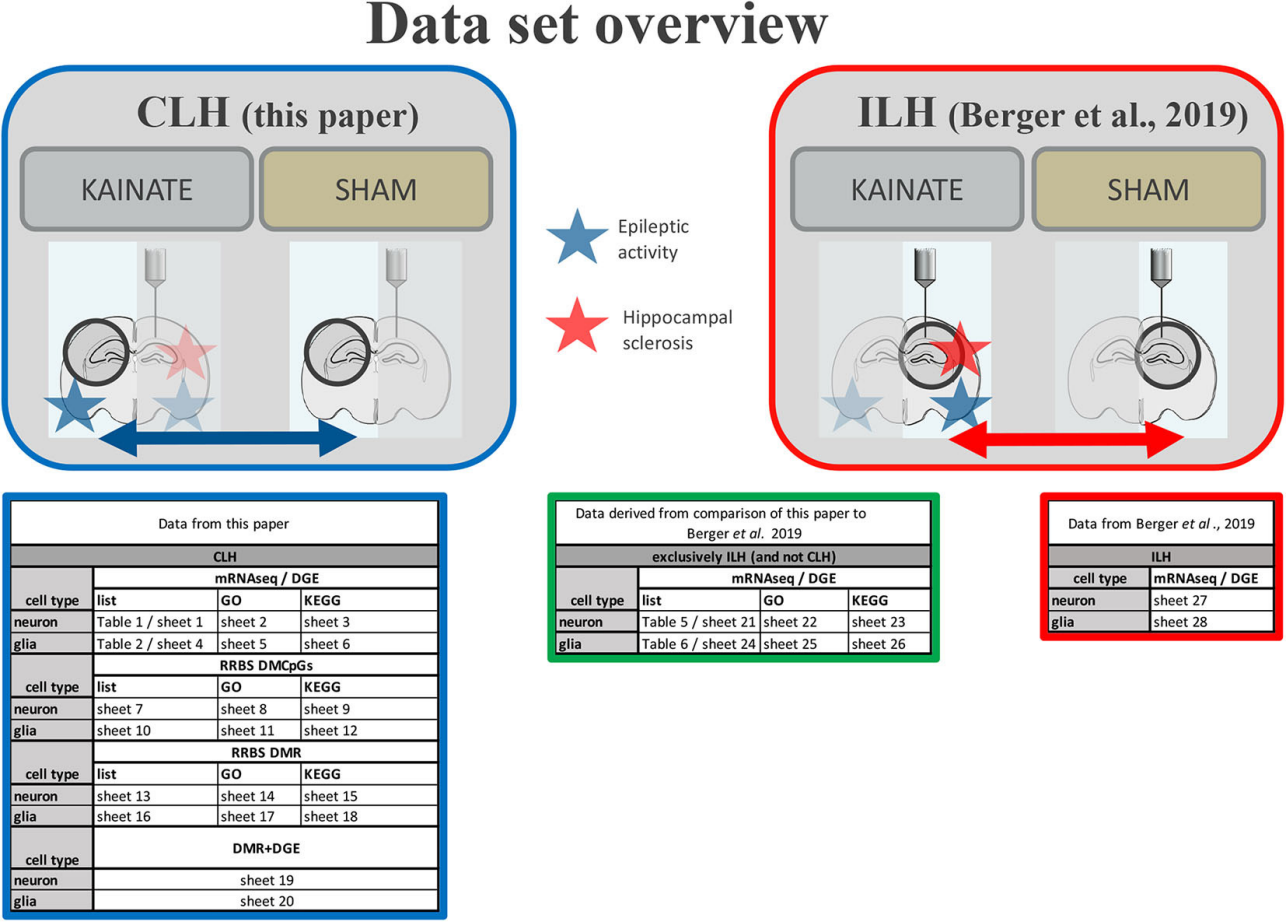
Data set overview. Overview of provided data in paper (Tables 1, 2, etc.) and Supplementary Table (sheets 1, 2, etc.) from the CLH (comparison of the contralateral hippocampi of the kainic acid group *vs*. sham group) and the ILH (comparison of the ipsilateral hippocampi of the kainic acid group *vs*. sham group as in Berger et al. (22).

A systematic overview of all data is given in Figure 2.

### Quality Control

The bisulfite conversion rates of the included samples were above 98% (Supplementary Figure 1). The multidimensional scaling plots for RRBS and mRNAseq (Supplementary Figure 2) clearly distinguished NeuN+ (referred to as neurons) and NeuN– (referred to as glia) fractions. As shown in the normalized mRNAseq plots (Supplementary Figure 3), the NeuN+ fraction was enriched for neuronal mRNA and the NeuN– fraction for glial mRNA. For further details regarding quality control of the differential gene expression and differential DNA methylation data, see Supplementary Document.

### Differential Gene Expression in the Hippocampus Contralateral to Kainate Injection

For the analysis of differentially expressed genes, mouse hippocampi contralateral to the kainate injection site were compared to the corresponding hippocampi of sham (saline)-treated animals 24 h post-injection. Differential gene expression was measured in neurons and glia separately. In neurons, 115 genes were upregulated and 16 downregulated (ratio of upregulated to downregulated genes, 7.19) (Table 1 and Supplementary Table, sheet 1). In glia, 74 genes were upregulated and 22 downregulated (ratio of upregulated to downregulated genes, 3.36) (Table 2 and Supplementary Table, sheet 4). We found an overlap of 27 genes differentially expressed in both neurons and glia. All of these were upregulated in both cell types. Comprehensive results from the GO term analysis and KEGG pathway analysis can be found in Supplementary Table (sheets 2, 3, 5, and 6). Selected results considered relevant for epileptogenesis are listed in Table 3 for neurons and Table 4 for glia cells.

**Table 1:** Differentially expressed genes in neurons in the contralateral hippocampus (CLH) at 24 h after kainate-induced status epilepticus.

**Gene symbol**	**logFC**	**FDR**	**Gene description**
**UPREGULATED GENES (*****N*** **=** **115)**			
**Sdc1**	**4.79**	**0.00**	**Syndecan 1**
Socs3	4.34	0.00	Suppressor of cytokine signaling 3
Cd1d1	3.99	0.00	CD1d1 antigen
**Col27a1**	**3.87**	**0.00**	**Collagen, type XXVII, alpha 1**
**Gal**	**6.07**	**0.00**	**Galanin**
Inhba	3.64	0.00	Inhibin beta-A
Lhfp	1.96	0.01	Lipoma HMGIC fusion partner
Ccn4	2.51	0.01	Cellular communication network factor 4
Tnc	2.27	0.01	Tenascin C
Megf11	2.17	0.01	Multiple EGF-like-domains 11
Nptx2	3.47	0.01	Neuronal pentraxin 2
Gipr	3.98	0.01	Gastric inhibitory polypeptide receptor
Pmepa1	2.54	0.01	Prostate transmembrane protein, androgen induced 1
**Parp3**	**3.47**	**0.01**	**Poly (ADP-ribose) polymerase family, member 3**
**Nedd9**	**1.72**	**0.02**	**Neural precursor cell expressed, developmentally downregulated gene 9**
Egr2	2.62	0.02	Early growth response 2
Fosb	3.36	0.02	FBJ osteosarcoma oncogene B
Crispld2	2.96	0.02	Cysteine-rich secretory protein LCCL domain containing 2
Pros1	2.71	0.03	Protein S (alpha)
Vim	3.14	0.03	Vimentin
Rgs4	2.58	0.03	Regulator of G-protein signaling 4
Prss23	2.39	0.03	Protease, serine 23
Ptgs2	2.89	0.04	Prostaglandin-endoperoxide synthase 2
Trh	6.51	0.04	Thyrotropin-releasing hormone
Sik1	2.08	0.04	Salt inducible kinase 1
Tll1	3.71	0.04	Tolloid-like
Fgl2	2.53	0.04	Fibrinogen-like protein 2
**Fos**	**2.99**	**0.04**	**FBJ osteosarcoma oncogene**
Adgrf4	2.41	0.05	Adhesion G protein-coupled receptor F4
Bag3	2.04	0.05	BCL2-associated athanogene 3
**Arc**	**2.12**	**0.06**	**Activity regulated cytoskeletal-associated protein**
Csrnp1	2.45	0.06	Cysteine–serine-rich nuclear protein 1
Angptl4	2.41	0.06	Angiopoietin-like 4
Ccl12	3.71	0.07	Chemokine (C-C motif) ligand 12
1700071M16Rik	1.92	0.07	1700071M16Rik
Fam129b	1.48	0.07	Family with sequence similarity 129, member B
Cemip2	1.68	0.07	Cell migration inducing hyaluronidase 2
Bmp3	2.11	0.07	Bone morphogenetic protein 3
Trib1	2.01	0.09	Tribbles pseudokinase 1
Rara	1.84	0.09	Retinoic acid receptor, alpha
Syndig1l	1.95	0.09	Synapse differentiation inducing 1 like
Dmp1	2.01	0.09	Dentin matrix protein 1
Cdk18	2.34	0.09	Cyclin-dependent kinase 18
Trib2	1.81	0.09	Tribbles pseudokinase 2 (Source: MGI symbol)
Gadd45g	2.14	0.09	Growth arrest and DNA-damage-inducible 45 gamma
Serinc2	1.99	0.09	Serine incorporator 2
Vgf	2.20	0.09	VGF nerve growth factor inducible
Tpbg	1.70	0.09	Trophoblast glycoprotein
Sulf1	1.39	0.10	Sulfatase 1
Srxn1	1.89	0.10	Sulfiredoxin 1 homolog
Acvr1c	2.22	0.10	Activin A receptor, type IC
Timp1	3.26	0.11	Tissue inhibitor of metalloproteinase 1
Ptx3	3.28	0.11	Pentraxin-related gene
Gpr3	1.93	0.11	G-protein-coupled receptor 3
Homer1	1.66	0.12	Homer scaffolding protein 1
Clcf1	2.67	0.12	Cardiotrophin-like cytokine factor 1
Cd1d2	3.31	0.12	CD1d2 antigen
Pappa	2.35	0.13	Pregnancy-associated plasma protein A
C2cd4b	2.39	0.14	C2 calcium-dependent domain containing 4B
Atf3	3.58	0.14	Activating transcription factor 3
Fndc9	2.91	0.14	Fibronectin type III domain containing 9
Acan	1.68	0.14	Aggrecan
Sbno2	1.99	0.16	Strawberry notch 2
Stk40	1.60	0.16	Serine/threonine kinase 40
Trip10	1.61	0.16	Thyroid hormone receptor interactor 10
Nfkbie	2.08	0.16	Nuclear factor of kappa light polypeptide gene enhancer in B cells inhibitor, epsilon
Anxa2	2.29	0.16	Annexin A2
Sphk1	2.41	0.16	Sphingosine kinase 1
Serpina3n	1.99	0.16	Serine (or cysteine) peptidase inhibitor, clade A, member 3N
Gfra1	1.63	0.16	Glial cell line derived neurotrophic factor family receptor alpha 1
Rasl11a	1.57	0.16	RAS-like, family 11, member A
Ier5	1.84	0.16	Immediate early response 5
Dgat2l6	3.38	0.16	Diacylglycerol *O*-acyltransferase 2-like 6
Hpgd	1.88	0.16	Hydroxyprostaglandin dehydrogenase 15 (NAD)
Pear1	3.10	0.16	Platelet endothelial aggregation receptor 1
Kif18a	1.63	0.16	Kinesin family member 18A
Prex1	1.98	0.16	Phosphatidylinositol-3,4,5-trisphosphate-dependent Rac exchange factor 1
Plpp4	1.97	0.16	Phospholipid phosphatase 4
Adamts6	1.66	0.16	A disintegrin-like and metallopeptidase (reprolysin type) with thrombospondin type 1 motif 6
**Dusp5**	**1.74**	**0.16**	**Dual specificity phosphatase 5**
Col5a3	1.99	0.18	Collagen, type V, alpha 3
Tnfrsf12a	2.04	0.18	Tumor necrosis factor receptor superfamily, member 12a
Ier2	2.09	0.18	Immediate early response 2
Tgfbr2	1.52	0.18	Transforming growth factor, beta receptor II
**Ptgs1**	**2.04**	**0.18**	**Prostaglandin-endoperoxide synthase 1**
Cdkn1a	2.41	0.18	Cyclin-dependent kinase inhibitor 1A
Cgref1	1.67	0.19	Cell growth regulator with EF hand domain 1
Arl4d	2.01	0.19	ADP-ribosylation factor-like 4D
Pipox	2.07	0.19	Pipecolic acid oxidase
Fosl2	1.62	0.19	Fos-like antigen 2
Pik3r6	1.76	0.20	Phosphoinositide-3-kinase regulatory subunit 5
Ccn1	2.26	0.20	Cellular communication network factor 1
Ltbp1	1.67	0.20	Latent transforming growth factor beta binding protein 1
Btg2	1.74	0.20	BTG anti-proliferation factor 2
Prlr	2.08	0.20	Prolactin receptor
Zfp36	2.22	0.20	Zinc finger protein 36
Efemp2	1.49	0.20	Epidermal growth factor-containing fibulin-like extracellular matrix protein 2
Rasa4	1.91	0.21	RAS p21 protein activator 4
Cd300lb	6.27	0.21	CD300 molecule like family member B
Sv2c	2.18	0.21	Synaptic vesicle glycoprotein 2c
Bdnf	1.78	0.21	Brain derived neurotrophic factor
Medag	2.15	0.21	Mesenteric estrogen-dependent adipogenesis
Mt1	2.12	0.21	Metallothionein 1
S100a10	1.93	0.21	S100 calcium binding protein A10
**Npy**	**2.23**	**0.23**	**Neuropeptide Y**
Notch1	1.80	0.24	Notch 1
Sstr2	2.15	0.24	Somatostatin receptor 2
Rbms1	1.40	0.24	RNA binding motif, single-stranded interacting protein 1
F2r	1.45	0.24	Coagulation factor II (thrombin) receptor
Chst5	1.77	0.24	Carbohydrate (*N*-acetylglucosamine 6-*O*) sulfotransferase 5
Msn	2.05	0.24	Moesin
**Mfap4**	**1.70**	**0.24**	**Microfibrillar-associated protein 4**
Adra1a	1.54	0.25	Adrenergic receptor, alpha 1a
Emp1	2.12	0.25	Epithelial membrane protein 1
Gadd45b	2.45	0.25	Growth arrest and DNA-damage-inducible 45 beta
**DOWNREGULATED GENES (*****N*** **=** **16)**			
Cxcl12	−1.88	0.04	Chemokine (C-X-C motif) ligand 12
Fbxl7	−1.43	0.06	F-box and leucine-rich repeat protein 7
Ogn	−2.54	0.06	Osteoglycin
Plk5	−2.54	0.10	Polo like kinase 5
Capn3	−2.00	0.11	Calpain 3
Atad2	−1.19	0.13	ATPase family, AAA domain containing 2
Pde7b	−1.51	0.16	Phosphodiesterase 7B
Prtg	−1.49	0.18	Protogenin
Per3	−1.45	0.19	Period circadian clock 3
Cys1	−1.52	0.19	Cystin 1
Smo	−1.76	0.20	Smoothened, frizzled class receptor
Gm12216	−1.50	0.21	Gm12216
Stxbp6	−1.47	0.24	Syntaxin binding protein 6
Cd34	−1.54	0.24	CD34 antigen
Plcg2	−1.54	0.24	Phospholipase C, gamma 2
Aqp11	−1.39	0.25	Aquaporin 11

*Differentially expressed genes in neurons in the contralateral hippocampus (FDR < 0.25)*.

*logFC, log fold change; FDR, false discovery rate*.

**Table 2:** Differentially expressed genes in glia in the contralateral hippocampus (CLH) at 24 h after kainate-induced status epilepticus.

**Gene symbol**	**logFC**	**FDR**	**Gene description**
**UPREGULATED GENES (*****N*** **=** **74)**			
**Socs3**	**5.55**	**0.00**	**Suppressor of cytokine signaling 3**
**Fos**	**5.82**	**0.00**	**FBJ osteosarcoma oncogene**
Serpina3n	4.19	0.00	Serine (or cysteine) peptidase inhibitor, clade A, member 3N
Cebpd	2.82	0.01	CCAAT/enhancer binding protein delta
Tnfrsf12a	3.85	0.01	Tumor necrosis factor receptor superfamily, member 12a
**Gadd45b**	**5.36**	**0.01**	**Growth arrest and DNA-damage-inducible 45 beta**
Emp1	4.30	0.01	Epithelial membrane protein 1
Ier5l	2.81	0.01	Immediate early response 5-like
S1pr3	3.68	0.01	Sphingosine-1-phosphate receptor 3
Egr1	4.17	0.01	Early growth response 1
Timp1	5.61	0.01	Tissue inhibitor of metalloproteinase 1
Egr2	3.11	0.01	Early growth response 2
**Tubb6**	**3.24**	**0.01**	**Tubulin, beta 6 class V**
Cd44	3.89	0.01	CD44 antigen
**Gadd45g**	**3.13**	**0.02**	**Growth arrest and DNA-damage-inducible 45 gamma**
Ccl2	3.28	0.02	Chemokine (C-C motif) ligand 2
Junb	3.68	0.02	Jun B proto-oncogene
Ptx3	5.09	0.02	Pentraxin related gene
Fosb	3.92	0.02	FBJ osteosarcoma oncogene B
Tm4sf1	4.35	0.02	Transmembrane 4 superfamily member 1
Sphk1	3.78	0.02	Sphingosine kinase 1
Gm3448	2.79	0.02	Predicted gene 3448
Ccl12	4.33	0.02	Chemokine (C-C motif) ligand 12
Vgf	3.00	0.02	VGF nerve growth factor inducible
Fstl4	2.60	0.03	Follistatin-like 4
Myc	2.84	0.03	Myelocytomatosis oncogene
Itga5	3.38	0.03	Integrin alpha 5 (fibronectin receptor alpha)
Cebpb	2.35	0.03	CCAAT/enhancer binding protein (C/EBP), beta
Arid5a	2.05	0.03	AT rich interactive domain 5A (MRF1-like)
Ier3	2.61	0.04	Immediate early response 3
Thbd	2.01	0.04	Thrombomodulin
Adamts1	2.86	0.05	A disintegrin-like and metallopeptidase with thrombospondin type 1 motif, 1
**Ecm1**	**2.09**	**0.05**	**Extracellular matrix protein 1**
Sbno2	2.62	0.05	Strawberry notch 2
Slc39a14	2.19	0.05	Solute carrier family 39 member 14
Hmga1b	2.22	0.05	High mobility group AT-hook 1B
Il1r1	1.90	0.06	Interleukin 1 receptor, type I
Klk9	3.35	0.06	Kallikrein related-peptidase 9
**Dusp5**	**2.25**	**0.07**	**Dual specificity phosphatase 5**
Ucn2	5.70	0.08	Urocortin 2
Gpr151	3.15	0.08	G protein-coupled receptor 151
Ier2	2.71	0.08	Immediate early response 2
Mafk	2.96	0.08	V-maf musculoaponeurotic fibrosarcoma oncogene family, protein K
Csrnp1	2.60	0.08	Cysteine–serine-rich nuclear protein 1
Itga7	1.74	0.09	Integrin alpha 7
Fosl2	2.05	0.10	Fos-like antigen 2
Bcl3	5.25	0.10	B cell leukemia/lymphoma 3
Atf3	4.24	0.12	Activating transcription factor 3
Fosl1	2.36	0.13	Fos-like antigen 1
Hmga1	2.49	0.13	High mobility group AT-hook 1
Loxl1	2.05	0.13	Lysyl oxidase-like 1
Mchr1	2.23	0.13	Melanin-concentrating hormone receptor 1
Kdm6b	1.71	0.13	KDM1 lysine (K)-specific demethylase 6B
Odc1	1.90	0.13	Ornithine decarboxylase, structural 1
Cd244a	2.96	0.13	CD244 molecule A
Msr1	3.59	0.15	Macrophage scavenger receptor 1
Sv2c	2.70	0.15	Synaptic vesicle glycoprotein 2c
Tma16	1.82	0.16	Translation machinery associated 16
C2cd4b	2.69	0.16	C2 calcium-dependent domain containing 4B
Egr4	2.41	0.16	Early growth response 4
Hspb1	3.46	0.17	Heat shock protein 1
Tnc	1.67	0.17	Tenascin C
Srxn1	1.92	0.17	Sulfiredoxin 1 homolog
Ahnak2	2.75	0.17	AHNAK nucleoprotein 2
Wwtr1	1.64	0.17	WW domain containing transcription regulator 1
Gpr3	2.00	0.18	G-protein coupled receptor 3
Zfp36	2.57	0.18	Zinc finger protein 36
Trib1	1.95	0.18	Tribbles pseudokinase 1
Il11	2.88	0.21	Interleukin 11
Rhoj	2.62	0.21	Ras homolog family member J
Hcar2	3.17	0.21	Hydroxycarboxylic acid receptor 2
Cdh22	2.02	0.24	Cadherin 22
Pvr	1.49	0.25	Poliovirus receptor
Slc7a1	1.23	0.25	Solute carrier family 7 member 1
**DOWNREGULATED GENES (*****N*** **=** **22)**			
Nat8f4	−2.30	0.01	*N*-acetyltransferase 8 (GCN5-related) family member 4
Hapln1	−2.80	0.03	Hyaluronan and proteoglycan link protein 1
Aifm3	−2.17	0.03	Apoptosis-inducing factor, mitochondrion-associated 3
Btbd17	−2.31	0.03	BTB (POZ) domain containing 17
Nwd1	−2.35	0.04	NACHT and WD repeat domain containing 1
Gdpd2	−2.40	0.06	Glycerophosphodiester phosphodiesterase domain containing 2
Slc2a5	−2.28	0.08	Solute carrier family 2 member 5
P2ry12	−2.63	0.09	Purinergic receptor P2Y, G-protein coupled 12
Gpr165	−2.10	0.10	G protein-coupled receptor 165
Tet1	−1.41	0.13	Tet methylcytosine dioxygenase 1
2900052N01Rik	−2.38	0.15	2900052N01Rik
Susd5	−2.04	0.15	Sushi domain containing 5
Maf	−1.49	0.17	Avian musculoaponeurotic fibrosarcoma oncogene homolog
Fn3k	−1.87	0.17	Fructosamine 3 kinase
Tmem255b	−2.02	0.17	Transmembrane protein 255B
Traf4	−1.75	0.17	TNF receptor associated factor 4
Fam228a	−1.73	0.17	Family with sequence similarity 228, member A
Paqr7	−1.94	0.20	Progestin and adipoQ receptor family member VII
Gpr34	−2.49	0.21	G protein-coupled receptor 34
Sowaha	−1.46	0.21	Sosondowah ankyrin repeat domain family member A
Phkg1	−1.77	0.25	Phosphorylase kinase gamma 1
Folh1	−2.08	0.25	Folate hydrolase 1

*Differentially expressed genes in glia in the contralateral hippocampus (FDR < 0.25)*.

*logFC, log fold change; FDR, false discovery rate*.

**Table 3:** Selection of relevant Gene Ontology (GO) and Kyoto Encyclopedia of Genes and Genomes (KEGG) terms of the differentially expressed genes in neurons in the contralateral hippocampus (CLH) at 24 h after kainate-induced status epilepticus.

	**Upregulated in neurons** ** (115 genes)**	**Downregulated in neurons** ** (16 genes)**
GO	Cell death	Positive regulation of calcium ion transport
	Regulation of cell proliferation	Axon guidance
	Cell communication	Vesicle organization
	MAPK cascade	Regulation of DNA-binding transcription factor activity
	Cell surface receptor signaling pathway	Axonogenesis
	Regulation of transcription, DNA-templated	Growth factor activity
	Vasculature development	Programmed cell death
	Inflammatory response	Blood vessel morphogenesis
KEGG	Cytokine-cytokine receptor interaction	Axon guidance
	cAMP signaling pathway	NF-kappa B signaling pathway
	ECM–receptor interaction	Leukocyte transendothelial migration
	IL-17 signaling pathway	–
	TGF-beta signaling pathway	–
	TNF signaling pathway	–
	MAPK signaling pathway	–
	p53 signaling pathway	–
	VEGF signaling pathway	–
	JAK-STAT signaling pathway	–

*Relevant GO and KEGG terms among the significantly differentially expressed genes (FDR < 0.25)*.

**Table 4:** Selection of relevant Gene Ontology (GO) and Kyoto Encyclopedia of Genes and Genomes (KEGG) terms of the differentially expressed genes in glia in the contralateral hippocampus (CLH) at 24 h after kainate-induced status epilepticus.

	**Upregulated in glia** ** (74 genes)**	**Downregulated in glia** ** (22 genes)**
GO	Regulation of angiogenesis	G protein-coupled nucleotide receptor activity
	Regulation of cell motility	Purinergic receptor activity
	Positive regulation of cell migration	Oxidation-reduction process
	Positive regulation of cell death	–
	Angiogenesis	–
	Regulation of interleukin-1 beta production	–
	Tumor necrosis factor production	–
	Neurogenesis	–
KEGG	TNF signaling pathway	–
	IL-17 signaling pathway	–
	ECM-receptor interaction	–
	MAPK signaling pathway	–
	Cytokine-cytokine receptor interaction	–
	Growth hormone synthesis, secretion and action	–
	JAK-STAT signaling pathway	–
	VEGF signaling pathway	–
	p53 signaling pathway	–
	PI3K-Akt signaling pathway	–
	NF-kappa B signaling pathway	–

*Relevant GO and KEGG terms amongst significantly differentially expressed genes (FDR < 0.25)*.

### Comparison of Differential Gene Expression Between the Contralateral and Ipsilateral Hippocampus

Comparison of differential gene expression in CLH to ILH (22) revealed similar numbers of differentially expressed genes for neurons, while there was a marked difference in the glia. In the neuronal fraction, 115 genes were upregulated in the CLH (Table 1 and Supplementary Table, sheet 1) while 132 genes were upregulated in the ILH (Supplementary Table, sheet 27). Sixteen genes were downregulated in the CLH (Table 1 and Supplementary Table, sheet 1) and 15 genes downregulated in the ILH (Supplementary Table, Sheet 27). In glia, only half of the number of genes were upregulated in the CLH (74 genes; Table 2 and Supplementary Table, sheet 4) compared with the ILH (147 genes; Supplementary Table, sheet 28). The difference was even more pronounced for the downregulated genes (22 in the CLH *vs*. 85 in the ILH).

#### Overlap of Differentially Expressed Genes in the Contralateral and Ipsilateral Hippocampus

**Figure 3:**
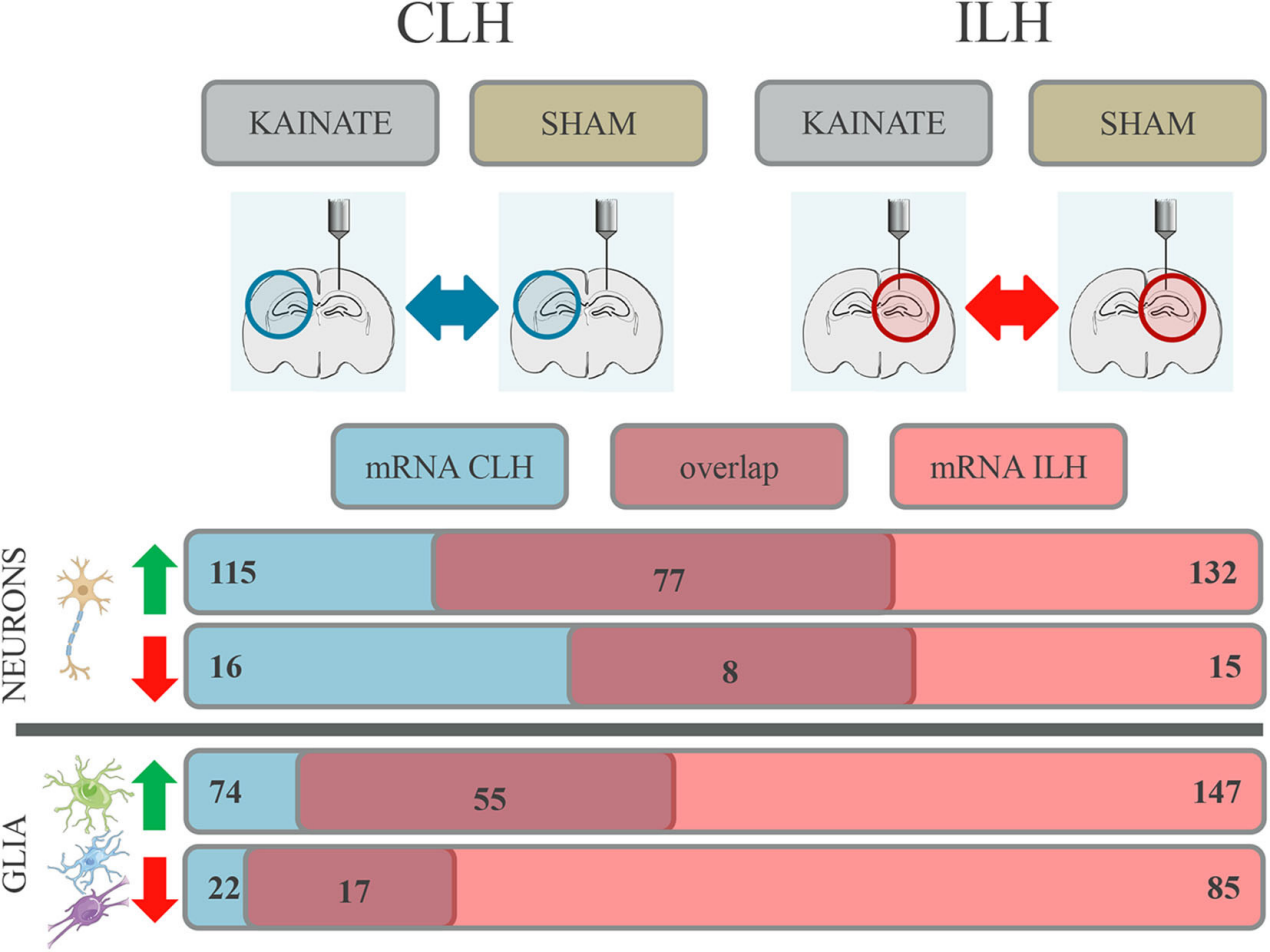
Differential gene expression with overlap in the contralateral (CLH) and ipsilateral hippocampus (ILH) in both neurons and glia at 24 h after kainate-induced status epilepticus. Number of genes in the neurons and glia showing differential expression in the CLH (numbers given in *blue box*), in the ILH (numbers given in *red box*), and in both hemispheres (numbers given in *dark red box*).

A comparison of genes differentially expressed in the CLH to those differentially expressed in the ILH (Supplementary Table, sheets 27 and 28) (22) revealed that a large number of differentially expressed genes coincided between CLH and ILH. This was the case both in the neurons and glia (Figure 3).

For neurons, 77 upregulated genes and eight downregulated genes were differentially expressed in both the CLH and ILH (Figure 3 and Supplementary Table, sheets 1 and 27). This constitutes a proportion of 65.22% (up) and 50.00% (down) of all the differentially regulated genes in the CLH. For glia, we found an overlap of 55 upregulated genes and an overlap of 17 downregulated genes between the CLH and ILH (Figure 3 and Supplementary Table, sheets 4 and 28). This translates to a proportion of 74.32% (up) and 77.27% (down) of all the differentially regulated genes in the CLH. All overlapping genes between the CLH and ILH showed concordant expression.

### Genes Exclusively Differentially Expressed in the Ipsilateral Hippocampus

In neurons, we found 55 genes to be upregulated and seven downregulated exclusively in the ILH (Table 5 and Supplementary Table, sheet 21). In glia, 92 genes were upregulated and 68 genes were downregulated exclusively in the ILH (Table 6 and Supplementary Table, sheet 24). The relevant GO and KEGG terms of these differentially expressed genes are listed in Tables 7, 8, and a detailed list of the GO and KEGG terms is to be found in Supplementary Table (sheets 22, 23, 25, and 26).

**Table 5:** Differentially expressed genes in neurons exclusively in the ILH (and not the CLH) at 24 h after kainate-induced status epilepticus.

**Gene symbol**	**logFC.ILH**	**FDR.ILH**	**Gene description**
**UPREGULATED GENES (*****N*** **=** **55; ILH: 132)**			
Hspa1a	5.46	0.01	Heat shock protein 1A
Mapk4	2.28	0.01	Mitogen-activated protein kinase 4
Hspa1b	4.82	0.02	Heat shock protein 1B
Pcdh11x	2.26	0.03	Protocadherin 11 X-linked
Pde6b	2.91	0.03	Phosphodiesterase 6B, cGMP, rod receptor, beta polypeptide
Wisp1	2.09	0.03	WNT1-inducible-signaling pathway protein 1
9330188P03Rik	3.35	0.03	RIKEN cDNA 9330188P03 gene
**Hspb1**	**4.00**	**0.03**	**Heat shock protein 1**
Lbh	3.01	0.03	Limb-bud and heart
Rrad	4.89	0.04	Ras-related associated with diabetes
4931440P22Rik	1.70	0.04	RIKEN cDNA 4931440P22 gene
Cyr61	2.90	0.04	Cysteine-rich angiogenic inducer 61
Zbtb46	1.58	0.05	Zinc finger and BTB domain containing 46
Bach1	1.72	0.05	BTB and CNC homology 1, basic leucine zipper transcription factor 1
Samd4	1.90	0.05	Sterile alpha motif domain containing 4
Npas4	3.24	0.06	Neuronal PAS domain protein 4
Adam19	1.62	0.08	A disintegrin and metallopeptidase domain 19 (meltrin beta)
Pim1	2.43	0.08	Proviral integration site 1
Mapkapk3	1.97	0.09	Mitogen-activated protein kinase-activated protein kinase 3
Cdh4	1.45	0.09	Cadherin 4
Kdm6b	1.57	0.09	KDM1 lysine (K)-specific demethylase 6B
**Spp1**	**3.14**	**0.09**	**Secreted phosphoprotein 1**
Sorcs3	2.28	0.09	Sortilin-related VPS10 domain containing receptor 3
Uck2	1.35	0.10	Uridine–cytidine kinase 2
Plce1	1.40	0.10	Phospholipase C, epsilon 1
Tgfb1i1	1.66	0.10	Transforming growth factor beta-1-induced transcript 1
Frrs1	1.87	0.12	Ferric-chelate reductase 1
Blnk	2.81	0.12	B cell linker
Rgs20	1.74	0.12	Regulator of G-protein signaling 20
Itprip	1.88	0.13	Inositol 1,4,5-triphosphate receptor interacting protein
Smad7	1.83	0.13	SMAD family member 7
Svil	1.52	0.13	Supervillin
**Mir132**	**3.39**	**0.15**	**MicroRNA 132**
Zdhhc22	1.85	0.17	Zinc finger, DHHC-type containing 22
Amotl1	1.71	0.18	Angiomotin-like 1
Serpina3i	2.75	0.18	Serine (or cysteine) peptidase inhibitor, clade A, member 3I
Ifit1	2.33	0.18	Interferon-induced protein with tetratricopeptide repeats 1
Kcnip3	1.67	0.18	Kv channel interacting protein 3, calsenilin
Odc1	1.57	0.18	Ornithine decarboxylase, structural 1
Igsf9b	2.27	0.18	Immunoglobulin superfamily, member 9B
Spred1	1.62	0.18	Sprouty protein with EVH-1 domain 1, related sequence
Samd11	2.19	0.19	Sterile alpha motif domain containing 11
Scd4	2.01	0.19	Stearoyl-coenzyme A desaturase 4
Dusp4	1.88	0.19	Dual specificity phosphatase 4
Tspan9	1.68	0.19	Tetraspanin 9
Eva1b	2.00	0.19	Eva-1 homolog B (*C. elegans*)
Btc	2.40	0.19	Betacellulin, epidermal growth factor family member
St8sia2	2.11	0.20	ST8 alpha-*N*-acetyl-neuraminide alpha-2,8-sialyltransferase 2
Tm4sf1	2.49	0.20	Transmembrane 4 superfamily member 1
Cdh22	1.77	0.20	Cadherin 22
Itga5	2.11	0.21	Integrin alpha 5 (fibronectin receptor alpha)
Mapk6	1.51	0.22	Mitogen-activated protein kinase 6
Egr4	1.91	0.23	Early growth response 4
Itpkc	1.84	0.23	Inositol 1,4,5-trisphosphate 3-kinase C
**Drd1**	**1.82**	**0.25**	**Dopamine receptor D1**
**DOWNREGULATED GENES (*****N*** **=** **7; ILH: 15)**			
Echdc2	−1.77	0.08	Enoyl coenzyme A hydratase domain containing 2
Cyp7b1	−2.28	0.10	Cytochrome P450, family 7, subfamily b, polypeptide 1
Gstm6	−1.57	0.16	Glutathione S-transferase, mu 6
Crlf1	−1.97	0.19	Cytokine receptor-like factor 1
Macrod1	−1.52	0.20	MACRO domain containing 1
Gm35339	−1.43	0.20	Predicted gene, 35339
6330420H09Rik	−2.15	0.22	RIKEN cDNA 6330420H09 gene

*Differentially expressed genes in neurons exclusively in the ipsilateral hippocampus (FDR < 0.25)*.

*logFC, log fold change; FDR, false discovery rate; ILH, ipsilateral hippocampus; CLH, contralateral hippocampus*.

**Table 6:** Differentially expressed genes in glia exclusively in the ILH (and not CLH) at 24 h after kainate-induced status epilepticus.

**Gene symbol**	**logFC.ILH**	**FDR.ILH**	**Gene description**
**UPREGULATED GENES (*****N*** **=** **92; ILH: 147)**			
Ch25h	5.14	0.00	Cholesterol 25-hydroxylase
Lilr4b	4.88	0.00	Leukocyte immunoglobulin-like receptor, subfamily B, member 4B
Calca	4.68	0.01	Calcitonin/calcitonin-related polypeptide, alpha
**Spp1**	**4.71**	**0.01**	**Secreted phosphoprotein 1**
Fn1	2.64	0.01	Fibronectin 1
Fgl2	2.97	0.01	Fibrinogen-like protein 2
Rasgef1c	3.04	0.01	RasGEF domain family, member 1C
**Ifit3**	**2.69**	**0.01**	**Interferon-induced protein with tetratricopeptide repeats 3**
**Iigp1**	**3.09**	**0.02**	**Interferon inducible GTPase 1**
Rasl11a	1.99	0.02	RAS-like, family 11, member A
Btc	3.29	0.02	Betacellulin, epidermal growth factor family member
Nptx2	2.91	0.03	Neuronal pentraxin 2
Adam8	3.04	0.03	A disintegrin and metallopeptidase domain 8
Inhba	2.61	0.03	Inhibin beta-A
Lilrb4a	4.02	0.03	Leukocyte immunoglobulin-like receptor, subfamily B, member 4A
Cd300lf	3.60	0.03	CD300 molecule like family member F
Cacng5	2.10	0.03	Calcium channel, voltage-dependent, gamma subunit 5
**Ifi204**	**4.09**	**0.03**	**Interferon activated gene 204**
Dab2	2.15	0.04	Disabled 2, mitogen-responsive phosphoprotein
Ifi207	3.11	0.04	Interferon activated gene 207
Parp3	2.74	0.04	Poly(ADP-ribose) polymerase family, member 3
Rasip1	2.30	0.05	Ras interacting protein 1
Lpl	1.88	0.06	Lipoprotein lipase
Tpbg	1.72	0.06	Trophoblast glycoprotein
Gcnt2	2.42	0.06	Glucosaminyl (*N*-acetyl) transferase 2, I-branching enzyme
Serpine1	3.97	0.06	Serine (or cysteine) peptidase inhibitor, clade E, member 1
Oasl2	2.47	0.06	2'-5' Oligoadenylate synthetase-like 2
**Ptgs2**	**2.43**	**0.07**	**Prostaglandin-endoperoxide synthase 2**
Slc10a6	3.88	0.07	Solute carrier family 10 (sodium/bile acid cotransporter family), member 6
Ahnak	1.95	0.07	AHNAK nucleoprotein (desmoyokin)
Nedd9	1.33	0.07	Neural precursor cell expressed, developmentally downregulated gene 9
Rai14	1.61	0.07	Retinoic acid induced 14
Layn	1.95	0.08	Layilin
Col16a1	2.51	0.08	Collagen, type XVI, alpha 1
Atp10a	2.07	0.08	ATPase, class V, type 10A
Gal	3.44	0.08	Galanin and GMAP prepropeptide
Mx1	3.40	0.08	MX dynamin-like GTPase 1
Irgm1	1.56	0.09	Immunity-related GTPase family M member 1
Gldn	3.04	0.09	Gliomedin
Cchcr1	1.58	0.09	Coiled-coil alpha-helical rod protein 1
Slc5a3	1.82	0.10	Solute carrier family 5 (inositol transporters), member 3
Socs2	1.76	0.10	Suppressor of cytokine signaling 2
**Il4ra**	**1.81**	**0.10**	**Interleukin 4 receptor, alpha**
Irf7	2.39	0.10	Interferon regulatory factor 7
Nlrc5	2.21	0.10	NLR family, CARD domain containing 5
Fgf18	2.32	0.11	Fibroblast growth factor 18
Ifit3b	2.41	0.11	Interferon-induced protein with tetratricopeptide repeats 3B
Strip2	1.74	0.12	Striatin interacting protein 2
Has2	3.19	0.12	Hyaluronan synthase 2
Mir212	4.52	0.12	MicroRNA 212
Flnc	3.71	0.12	Filamin C, gamma
Map3k6	2.39	0.12	Mitogen-activated protein kinase kinase kinase 6
Timeless	1.39	0.12	Timeless circadian clock 1
Snhg15	1.56	0.13	Small nucleolar RNA host gene 15
Mamstr	2.09	0.14	MEF2 activating motif and SAP domain containing transcriptional regulator
Clcf1	2.36	0.14	Cardiotrophin-like cytokine factor 1
**Bdnf**	**1.81**	**0.14**	**Brain-derived neurotrophic factor**
Rnf138rt1	5.32	0.15	Ring finger protein 138, retrogene 1
Slfn10-ps	2.78	0.16	Schlafen 10, pseudogene
Amotl1	1.65	0.16	Angiomotin-like 1
**Mir132**	**3.30**	**0.17**	**MicroRNA 132**
Serpina3i	2.64	0.17	Serine (or cysteine) peptidase inhibitor, clade A, member 3I
Hmox1	1.87	0.17	Heme oxygenase 1
Lrtm2	1.62	0.18	Leucine-rich repeats and transmembrane domains 2
Spred3	1.72	0.18	Sprouty-related EVH1 domain containing 3
Vmn1r15	6.73	0.18	Vomeronasal 1 receptor 15
Rtp4	1.91	0.18	Receptor transporter protein 4
Rnf125	2.28	0.18	Ring finger protein 125
Slfn2	2.93	0.18	Schlafen 2
Piezo2	1.68	0.19	Piezo-type mechanosensitive ion channel component 2
Anxa2	2.01	0.19	Annexin A2
Gpd1	1.68	0.19	Glycerol-3-phosphate dehydrogenase 1 (soluble)
Cyr61	2.08	0.19	Cysteine-rich angiogenic inducer 61
Plaur	2.39	0.19	Plasminogen activator, urokinase receptor
Ifit1	2.11	0.20	Interferon-induced protein with tetratricopeptide repeats 1
Itga2b	1.93	0.20	Integrin alpha 2b
Fgfr4	2.25	0.20	Fibroblast growth factor receptor 4
Bst2	2.06	0.20	Bone marrow stromal cell antigen 2
Gm6225	2.35	0.21	Predicted gene 6225
Cbln4	1.60	0.21	Cerebellin 4 precursor protein
Serpina3m	2.79	0.22	Serine (or cysteine) peptidase inhibitor, clade A, member 3M
Akap12	1.34	0.22	A kinase (PRKA) anchor protein (gravin) 12
Sdc1	1.59	0.22	Syndecan 1
Ndst1	1.59	0.22	*N*-deacetylase/*N*-sulfotransferase (heparan glucosaminyl) 1
Npas4	2.45	0.22	Neuronal PAS domain protein 4
Tspan4	1.89	0.23	Tetraspanin 4
Klk6	2.76	0.23	Kallikrein related-peptidase 6
**Cxcl10**	**2.90**	**0.23**	**Chemokine (C-X-C motif) ligand 10**
Col7a1	1.75	0.23	Collagen, type VII, alpha 1
Plce1	1.17	0.24	Phospholipase C, epsilon 1
Peak1	1.41	0.24	Pseudopodium-enriched atypical kinase 1
Itga1	1.36	0.25	Integrin alpha 1
**DOWNREGULATED GENES (*****N*** **=** **68; ILH: 85)**			
Pcx	−2.11	0.02	Pyruvate carboxylase
Shroom2	−2.28	0.03	Shroom family member 2
Gpr12	−2.22	0.04	G-protein-coupled receptor 12
Ccdc13	−1.80	0.05	Coiled-coil domain containing 13
Cygb	−1.88	0.05	Cytoglobin
Ankub1	−2.23	0.06	Ankrin repeat and ubiquitin domain containing 1
Siglech	−2.16	0.06	Sialic acid binding Ig-like lectin H
Itpka	−1.70	0.06	Inositol 1,4,5-trisphosphate 3-kinase A
Hpca	−1.84	0.07	Hippocalcin
Ppp1r1b	−1.75	0.08	Protein phosphatase 1, regulatory inhibitor subunit 1B
Nkain4	−2.55	0.08	Na^+^/K^+^ transporting ATPase interacting 4
Kctd4	−2.09	0.08	Potassium channel tetramerization domain containing 4
Gstm6	−1.67	0.08	Glutathione S-transferase, mu 6
Shisa8	−2.21	0.09	Shisa family member 8
2810468N07Rik	−2.22	0.09	RIKEN cDNA 2810468N07 gene
Abca9	−1.97	0.09	ATP-binding cassette, sub-family A (ABC1), member 9
Chn1	−1.71	0.10	Chimerin 1
Ntsr2	−2.14	0.11	Neurotensin receptor 2
Myh14	−1.76	0.11	Myosin, heavy polypeptide 14
Fam234a	−1.77	0.11	Family with sequence similarity 234, member A
Faah	−1.50	0.12	Fatty acid amide hydrolase
Tppp3	−1.76	0.12	Tubulin polymerization-promoting protein family member 3
Abca6	−1.46	0.12	ATP-binding cassette, sub-family A (ABC1), member 6
Gnai1	−1.90	0.13	Guanine nucleotide binding protein (G protein), alpha inhibiting 1
Cfap100	−1.48	0.14	Cilia and flagella associated protein 100
**Grm3**	**−2.01**	**0.14**	**Glutamate receptor, metabotropic 3**
Phgdh	−1.66	0.15	3-Phosphoglycerate dehydrogenase
Selplg	−2.14	0.15	Selectin, platelet (p-selectin) ligand
Epn2	−1.61	0.17	Epsin 2
Rlbp1	−1.78	0.18	Retinaldehyde binding protein 1
Pantr1	−1.72	0.18	POU domain, class 3, transcription factor 3 adjacent non-coding transcript 1
Plk5	−2.14	0.18	Polo-like kinase 5
Nat8f1	−1.91	0.18	*N*-acetyltransferase 8 (GCN5-related) family member 1
1700066M21Rik	−1.65	0.18	RIKEN cDNA 1700066M21 gene
Adi1	−1.61	0.18	Acireductone dioxygenase 1
Tmem191c	−1.45	0.18	Transmembrane protein 191C
Gmnc	−2.55	0.18	Geminin coiled-coil domain containing
Zfp763	−1.51	0.18	Zinc finger protein 763
Slc25a18	−1.79	0.19	Solute carrier family 25 (mitochondrial carrier), member 18
Hhip	−2.01	0.19	Hedgehog-interacting protein
Calb1	−1.51	0.19	Calbindin 1
Chst5	−1.74	0.19	Carbohydrate (*N*-acetylglucosamine 6-*O*) sulfotransferase 5
Trim59	−2.18	0.19	Tripartite motif-containing 59
Olfml1	−2.24	0.19	Olfactomedin-like 1
Mturn	−1.41	0.19	Maturin, neural progenitor differentiation regulator homolog (*Xenopus*)
Gstm1	−1.80	0.19	Glutathione S-transferase, mu 1
Enho	−1.63	0.19	Energy homeostasis associated
Prodh	−1.86	0.19	Proline dehydrogenase
Slc27a1	−1.71	0.19	Solute carrier family 27 (fatty acid transporter), member 1
Pacsin3	−1.44	0.19	Protein kinase C and casein kinase substrate in neurons 3
Htr1a	−1.95	0.19	5-Hydroxytryptamine (serotonin) receptor 1A
Dll3	−1.72	0.19	Delta-like canonical Notch ligand 3
Map6d1	−1.60	0.19	MAP6 domain containing 1
Prrg1	−1.61	0.19	Proline-rich Gla (G-carboxyglutamic acid) 1
Carns1	−1.88	0.20	Carnosine synthase 1
Tle2	−1.48	0.20	Transducin-like enhancer of split 2
Macrod1	−1.45	0.20	MACRO domain containing 1
Nrgn	−1.51	0.20	Neurogranin
Plin3	−2.18	0.21	Perilipin 3
Grhpr	−1.38	0.21	Glyoxylate reductase/hydroxypyruvate reductase
Sult1a1	−2.19	0.21	Sulfotransferase family 1A, phenol-preferring, member 1
Pls1	−1.58	0.22	Plastin 1 (I-isoform)
Lin7b	−1.69	0.22	Lin-7 homolog B (*C. elegans*)
Armh4	−1.53	0.22	Armadillo-like helical domain containing 4
Panx2	−1.33	0.23	Pannexin 2
Appl2	−1.76	0.23	Adaptor protein, phosphotyrosine interaction, PH domain and leucine zipper containing 2
Grhl1	−1.01	0.23	Grainyhead-like transcription factor 1
Pigz	−1.71	0.24	Phosphatidylinositol glycan anchor biosynthesis, class Z

*Differentially expressed genes in glia exclusively in the ipsilateral hippocampus (FDR < 0.25)*.

*logFC, log fold change; FDR, false discovery rate; ILH, ipsilateral hippocampus; CLH, contralateral hippocampus*.

**Table 7:** Selection of relevant Gene Ontology (GO) and Kyoto Encyclopedia of Genes and Genomes (KEGG) terms of the differentially expressed genes in neurons in the ipsilateral hippocampus (but not in the contralateral hippocampus) at 24 h after kainate-induced status epilepticus.

	**Upregulated in neurons** ** (55 genes)**	**Downregulated in neurons** ** (7 genes)**
GO	Ion binding	Monocarboxylic acid metabolic process
	MAP kinase activity	Oxidation-reduction process
	Protein phosphorylation	Carboxylic acid metabolic process
	Cell communication	Positive regulation of cell proliferation
	Regulation of spindle assembly	Lipid metabolic process
	Protein serine/threonine kinase inhibitor activity	–
	Regulation of synaptic plasticity	–
	Cell–cell adhesion	–
KEGG	MAPK signaling pathway	–
	VEGF signaling pathway	–
	Calcium signaling pathway	–
	Inositol phosphate metabolism	–
	Antigen processing and presentation	–
	ECM–receptor interaction	–
	IL-17 signaling pathway	–
	Phosphatidylinositol signaling system	–

*Relevant GO and KEGG terms among the significantly differentially expressed genes (FDR < 0.25)*.

**Table 8:** Selection of relevant Gene Ontology (GO) and Kyoto Encyclopedia of Genes and Genomes (KEGG) terms of the differentially expressed genes in glia in the ipsilateral hippocampus (but not in the contralateral hippocampus) at 24 h after kainate-induced status epilepticus.

	**Upregulated in glia** ** (92 genes)**	**Downregulated in glia** ** (68 genes)**
GO	Cell differentiation	Dendrite
	Immune system process	Modulation of chemical synaptic transmission
	Positive regulation of cell differentiation	Calcium channel regulator activity
	Positive regulation of cell motility	Regulation of synaptic plasticity
	Extracellular space	Glutamine family amino acid metabolic process
	Vasculature development	Myelin sheath
	Regulation of programmed cell death	Glutamate receptor signaling pathway
	–	GABA-ergic synapse
KEGG	ECM–receptor interaction	Glutathione metabolism
	PI3K–Akt signaling pathway	cAMP signaling pathway
	MAPK signaling pathway	Pyruvate metabolism
	Toll-like receptor signaling pathway	Glycine, serine and threonine metabolism
	Cytokine–cytokine receptor interaction	ABC transporters
	IL-17 signaling pathway	Glutamatergic synapse
	Complement and coagulation cascades	–

*Relevant GO and KEGG terms among the significantly differentially expressed genes (FDR < 0.25)*.

### Differential Methylation in the Hippocampus Contralateral to Kainate Injection

**Figure 4:**
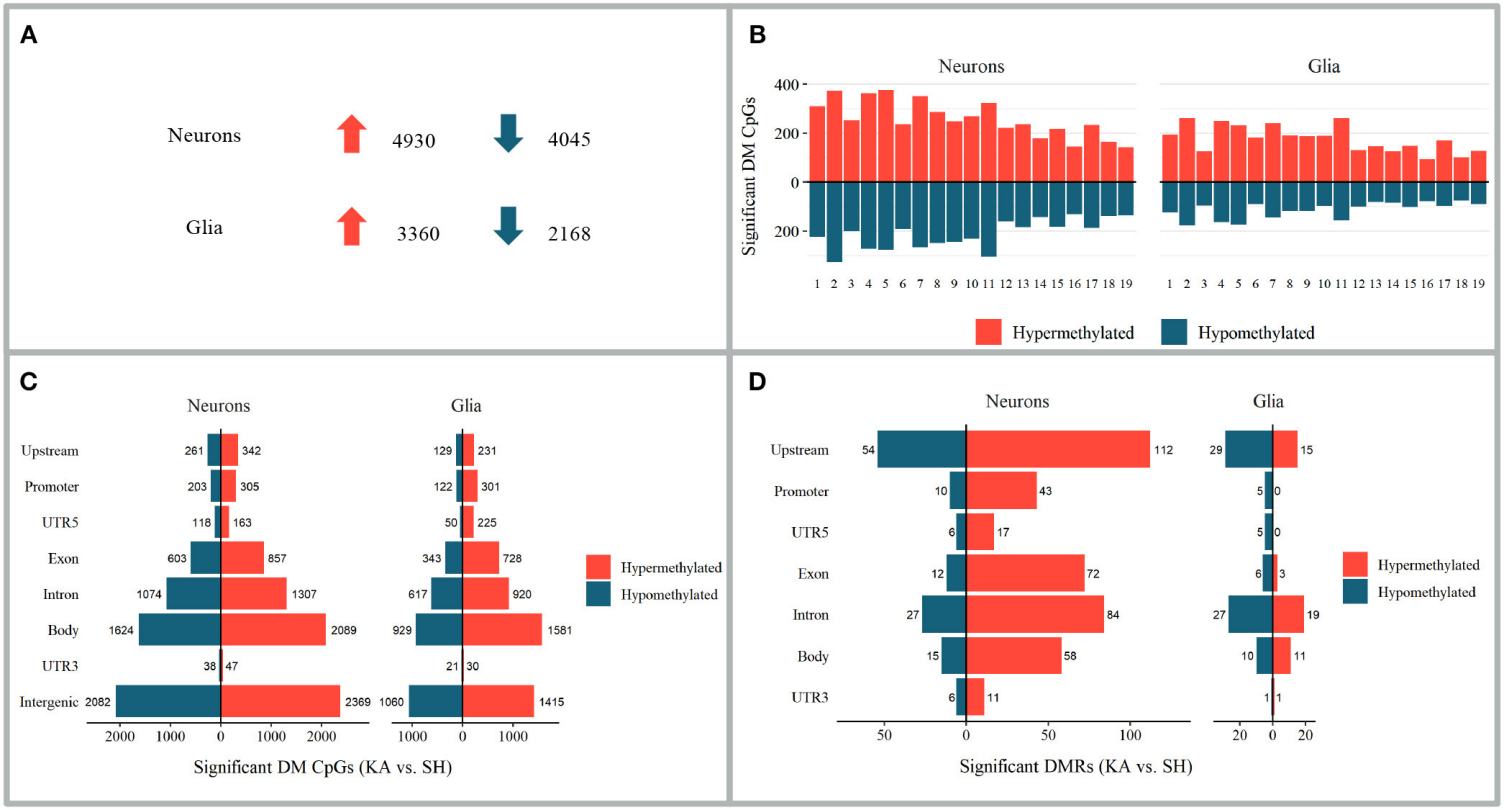
Alterations in DNA methylation 24 h after kainate-induced status epilepticus in hippocampi contralateral to injection site. **(A**–**C)** Differentially methylated CpGs of the kainic acid group *vs*. the sham group. **(A)** Number of differentially methylated CpGs in neurons and glia; *upward arrow* implies hypermethylation and *downward arrow* hypomethylation. **(B)** Chromosomal distribution of differentially methylated CpGs. **(C)** Distribution of differentially methylated CpGs among genomic features. **(D)** Distribution of differentially methylated regions among genomic features.

Differentially methylated CpGs were analyzed comparing left (contralateral) hippocampi of the kainic acid group to the sham group at 24 h after status epilepticus induction. For an overview of the number and distribution of the differentially methylated sites and the differentially methylated regions, see Figure 4. For a detailed list of the differentially methylated CpGs, differentially methylated regions, and the associated GO and KEGG terms, see Supplementary Table (differentially methylated CpGs: sheets 7 and 10; differentially methylated regions: sheets 13 and 16; differentially methylated CpGs GO: sheets 8 and 11; differentially methylated CpGs KEGG: sheets 9 and 12; differentially methylated regions GO: sheets 14 and 17; differentially methylated regions KEGG: sheets 15 and 18).

### Overlap of Differentially Methylated CpGs Between Neurons and Glia

Ten CpG sites (0.12% of all differentially methylated CpGs in the CLH) were hypermethylated and six CpG sites (0.09%) hypomethylated in both neurons and glia. Twenty-one CpGs (0.18%) were hypermethylated in neurons and hypomethylated in glia and 13 hypomethylated in neurons and hypermethylated in glia (0.18%).

### Association Between Differential Methylation and Differential Gene Expression in the Contralateral Hippocampus

In order to investigate a possible statistical association between differential DNA methylation and differential gene expression in the CLH, significantly differentially methylated regions and differentially expressed genes were aligned. No general trend in the association between the differentially methylated regions (upstream, promoter, UTR5, exon, intron, gene body, and UTR3) and differential gene expression was found (see figures in Supplementary Table, sheets 19 and 20), but significant alterations in DNA methylation and gene expression coincided at 11 genomic loci for neurons and four genomic loci for glia (Supplementary Table, sheets 19 and 20).

### Differential DNA Methylation, and Association of Differential Methylation With Differential Gene Expression, in the Contralateral Hippocampus Compared With the Ipsilateral Hippocampus

Only a fragment of the differentially methylated CpGs and the differentially methylated regions overlapped between the CLH and ILH (22). Of all the differentially methylated CpGs in neurons in the CLH, 48 (0.44% of all the differentially methylated CpGs in neurons) were also differentially methylated in neurons in the ILH (22 hypermethylated and 26 hypomethylated). In glia, seven differentially methylated CpGs (0.11%) were differentially methylated in both the CLH and ILH (four hypermethylated and three hypomethylated). Regarding differentially methylated regions, 17 were overlapping between the ILH and CLH in neurons (16 hypermethylated and one hypomethylated) and two (both hypermethylated) in glia. Some of these overlapping differentially methylated CpGs and differentially methylated regions were linked to genes with epilepsy- and DNA methylation-relevant functions like TGF-beta signaling, DNA methyltransferase activity, or angiogenesis, but none of these overlapped with the differentially expressed genes in the ipsilateral or contralateral hippocampus (Supplementary Table).

Only one gene, *Spp1*, had an association between differential DNA methylation and differential gene expression in both the CLH and ILH (neurons). *Spp1* was upregulated in both CLH and ILH. This coincided with upstream and promoter hypermethylation in the CLH and upstream and promoter hypomethylation in the ILH. No overlaps for differential DNA methylation and differential gene expression associations were found for glia when comparing CLH to ILH (Supplementary Table) (22).

## Discussion

In this study, we investigate alterations in gene expression and DNA methylation in glia and neurons in mouse hippocampi contralateral to intracortical kainic acid application. We found fulminant changes of both the gene expression and DNA methylation in glia and neurons in the CLH at 24 h after kainate-induced status epilepticus.

Based on our findings, we will discuss possible beneficial and detrimental responses to epileptic activity in the CLH. We will further illuminate potential genetic targets relevant to hippocampal sclerosis by comparing alterations in gene expression in the CLH to gene expression in the ILH. Lastly, we will discuss DNA methylation and its role for gene expression regulation in early epileptogenesis.

### Differential Gene Expression in the Contralateral Hippocampus Only Exposed to Epileptic Activity

We found that differential gene expression in the contralateral hippocampus at 24 h after kainate-induced status epilepticus mainly occurs cell-specific, with only a minor overlap of genes differentially expressed in the neurons and glia. This may reflect the complementary characteristic of neuron–glia interactions in epilepsy (4) and is comparable to our previous findings from cell-specific gene expression in the ipsilateral hippocampus (22).

In the CLH, the primary factor affecting differential gene expression is related to epileptic activity upon status epilepticus (Figure 1) (11). Differentially expressed genes comprise diverse inflammatory responses, synaptic signaling, and DNA methylation machinery in both neurons and glia (Tables 3, 4). Many of the gene expression changes seen in the CLH (lacking hippocampal sclerosis) overlap with our previous findings from the ILH [comprising hippocampal sclerosis at chronic time points: Supplementary Table 27 and 28 (22)]. This may appear unexpected since the CLH does not show morphological changes as seen in the ILH like reactive gliosis and neuronal death. A previous study on the ipsilateral and contralateral gene expression changes in a unilateral kainic acid epilepsy model also found a large overlap of the differentially expressed genes in the ipsilateral and contralateral hippocampus (43). The authors created different subsets of genes in order to distinguish between the effect of the kainic acid-induced lesion and epileptic seizures. A comparison of our data obtained at an early time point of epileptogenesis from the CLH to these results at a chronic stage of epileptogenesis reveals several genes overlapping with the “seizure” gene set (neurons: *Gal, Fos, Parp3, Nedd9, Mfap4, Dusp5, Col27a1, Sdc1, Ptgs1*, and *Arc*; glia: *Tubb6, Fos, Ecm1*, and *Dusp5*).

We further find a great degree of overlap between the gene expression changes seen in the CLH with other studies of various animal models for epilepsy (29, 44), gene expression material from the resected hippocampi of temporal lobe epilepsy (TLE) patients (45), and even with genomic data from animal models of reactive gliosis (46).

If the gene expression response in the CLH is so similar to both the ILH (with morphological alterations such as neuronal cell death and reactive gliosis 3 months after status epilepticus initiation or earlier) (11) and diverse models of epilepsy and reactive gliosis, why does the CLH not develop comparable morphological changes? The question whether epileptic activity can lead to morphological changes has long been a matter of controversial debate and is, to date, unanswered. While several previous studies claim that seizures mediate epileptogenic effects (47, 48), others postulate that seizures *per se* do not promote epileptogenesis (35, 49).

A hypothesis as to why the CLH remains free of hippocampal sclerosis may be that it is exposed to fewer detrimental or a larger number of beneficial effects, or both. As for fewer detrimental effects, one apparent characteristic in the CLH is the significantly lower number of glial genes up- and downregulated compared to the ILH. Only half the number of genes are up- and only a quarter of the number of genes are downregulated compared to the ILH. With several glial genes coding for pro-inflammatory pathways (Supplementary Table, sheets 4–6), this less pronounced glial activation in the contralateral hippocampus may be related to the lack of morphological changes characteristically observed at later time points. With regard to the gene expression changes with possible beneficial effects in the CLH, we find several seizure-alleviating and even potential anti-epileptic genes and pathways upregulated. Within the glial genes in the CLH, more genes overlapped with a gene set previously related to a “beneficial” type of astrocyte (A2) than with the gene set of “detrimental” astrocytes (A1) (46), possibly representing a glia-mediated endogenic anti-epileptogenic process in early epileptogenesis. Other epileptic activity-induced genes with seizure-alleviating or potentially even anti-epileptogenic effects include *Gal, Socs3*, and *NPY*. GAL (galanin) has previously been shown to exhibit anti-seizure effects and comprises potential anti-epileptogenic qualities (50). The gene expression levels of *Galanin* are elevated in neurons in the CLH, possibly revealing epileptic activity-related homeostatic effects. Further, we find the gene expression levels of NPY (neuropeptide Y), a neuropeptide recently successfully shown to attenuate seizures in slices of medication-refractory TLE (51), elevated in neurons in the CLH. Lastly, we find elevated levels of SOCS3 in glia in the CLH. *Socs3* codes for the suppressor of cytokine signaling 3 protein. This protein reduces the pro-inflammatory responses of, among others, IL-6, IFN, IL2, Il12, and NfkB signaling pathways and reduces astrocytic chemokine production (52). Thus, *Socs3* expression potentially represents another example of an endogenic reaction aiming at reducing the detrimental effects of seizures.

In sum, we speculate that anti-epileptogenic effects may outweigh pro-epileptogenic effects and thus prevent morphological alterations like neuronal death and reactive gliosis in the CLH. In fact, we find a higher fraction of GO terms anticipating anti-epileptogenic effects like “neurogenesis” (glia) and a lower number of GO terms indicating pro-epileptogenic qualities like “negative regulation of neuronal death” (neurons) in the CLH (Supplementary Table, sheets 2 and 5) compared to the ILH (22).

### Potential Upstream Targets of Hippocampal Sclerosis and Epileptogenesis

If one were to speculate which genes in our ipsilateral and contralateral findings in early epileptogenesis were most likely potential candidate genes driving hippocampal sclerosis and epileptogenesis, one could hypothesize that these would have to be exclusively found on the list of differentially expressed genes in the ILH. Featuring morphological changes like reactive gliosis and neuronal death, the ILH is associated with epileptogenesis (Figure 1).

For neurons, genes only differentially expressed in the ILH comprise pathways within various inflammatory responses and epilepsy-relevant genes like *Mir132* (53) and *Drd1* (54) (Table 5 and Supplementary Table, sheet 21). In glia, genes upregulated in the ILH but not in the CLH include several interferon- and interleukin-associated genes like *Ifit3, Iigp1, Ifi204*, and *Il4ra*, other inflammatory genes previously associated with epilepsy like *Ptgs2* (Cox2) (55), and epilepsy-related genes like *Bdnf* (56) and *Mir132* (53) (Table 6 and Supplementary Table, sheet 24). Downregulated genes in glia involve, among others, *Grm3*, a gene encoding for the metabotropic glutamate receptor 3, previously shown to be downregulated in experimental and human mTLE (57).

Within these genes exclusively differentially expressed in the ILH (and not CLH), one could check for overlaps with the top target genes in the reactive gliosis gene set mentioned earlier. Glial *CxCl10* and *Ptgs2* (*Cox2*) are exclusively differentially expressed in both the ILH (22) and in a previous genomic analysis of reactive gliosis (46). CXCL10, a chemokine elevated in various central nervous system (CNS) pathologies like Alzheimer's disease (58), multiple sclerosis (59), and Rasmussen encephalitis (60), has been shown to elicit elevated neuronal excitability after acute (61) and chronic exposure (62). Produced in astrocytes (63), it mediates neuronal death *via* Ca^2+^-dependent apoptosis (64). *Ptgs2*, coding for COX2, a cyclooxygenase exerting pro-epileptogenic effects in epileptogenesis (55), represents another potential glial upstream target for anti-epileptogenic intervention. These findings are in line with previous studies on the importance of glia-driven inflammatory pathways in epileptogenesis (4, 65).

As mentioned, the number of genes differentially expressed by the glia in the ILH are significantly higher than those in the CLH. This possibly indicates a more pronounced glial reaction triggered by the combination of epileptic activity and kainate in the ILH. This is supported by the notion of previously reported glial responses to kainic acid injection (66). In the intracortical model of mTLE-HS, the effects of epileptic activity and kainate are difficult to disentangle. Both kainate (67–69) and epileptic activity (47, 48, 70) can exert cytotoxic effects that, in combination, might be potentiated (71, 72). A previous genomic analysis of the ipsilateral and contralateral hippocampi of kainate-injected rats in chronic epilepsy (43) created a “kainic acid” gene set—a list of genes presumably induced by kainic acid. We find a surprisingly small overlap of these “kainic acid genes” with our data (exclusively ILH: *Spp1* and *Hspb1* in neurons and *Spp1* in the glia), possibly indicating that the singular effect of kainate may not be of primordial importance for downstream effects like hippocampal sclerosis and epileptogenesis after all (for restrictions in interpretability, see *Limitations*). Further, our goal was to identify upstream gene expression alterations possibly leading to hippocampal sclerosis, and as such, the exact cause of these alterations may be of secondary importance as long as they lead to epileptogenesis-relevant hallmarks.

### DNA Methylation and Its Role for Gene Expression in Early Epileptogenesis

In line with previous studies (22, 73, 74), DNA methylation occurs mainly in a cell-specific manner in the CLH. Regarding the methylation of singular CpG sites, hypermethylation slightly outweighs hypomethylation in both neurons and glia, both with regards to differentially methylated CpGs in total and differentially methylated CpGs within genomic regions. This trend is similar to the DNA methylation dynamics observed at 24 h in the ILH (22) and to previous data from DNA methylation alterations in a rat model of chronic epilepsy (75). Differentially methylated regions were mostly hypermethylated in neurons and hypomethylated in glia. This represents a near inversion of the methylation pattern of the differentially methylated regions in the ILH, where most differentially methylated regions in neurons were hypomethylated and most differentially methylated regions in the glia were hypermethylated (22). Previous studies of epilepsy-related DNA methylation in acute phases of epilepsy in animal models found no general trend toward hyper- or hypomethylation (30) or a tendency toward hypomethylation (76).

One possible reason for the higher ratio of hypomethylated differentially methylated regions in glia in the CLH is the higher levels of gene expression of *Gadd 45b* and *Gadd 45g*, which both are capable of DNA demethylation (77). In the CLH, significant alterations of differential DNA methylation and differential gene expression coincided at several genomic loci (Supplementary Table, sheets 19 and 20), e.g., at epilepsy-relevant genes like *Spp1* (78) in neurons and *Atf3* (79) in glia. Differential gene expression and differential DNA methylation coincide at epilepsy-related loci in both the CLH and ILH, yet the overlap of differential methylation between the CLH and ILH is marginal. There are no genomic loci in both the CLH and ILH at which differential DNA methylation and differential gene expression coincide in both hippocampi. While several previous studies revealed various associations between DNA methylation and gene expression in epilepsy (28, 29, 75), more recent studies have claimed a more restricted importance of DNA methylation for gene expression in epilepsy (80). The general role of DNA methylation for the regulation of gene expression appears to be highly tissue- and context-specific (81) and may not be the primary factor determining gene expression in early epileptogenesis. Thus, how changes in DNA methylation are related to differential gene expression in early epileptogenesis remains unclear.

### Limitations

Considered a solid marker of mature neurons (82, 83), NeuN (Rbfox3) may not stain all CNS neurons (84). As such, the NeuN– fraction (referred to as glia) may, apart from astrocytes, oligodendrocytes, and microglia, contain a minor fraction of non-glial cells (e.g., endothelial cells, pericytes, and neurons) (84–86).

At steady state, RNA sequencing (RNAseq) is a solid approach for the estimation of protein abundance, and as such, biological function, yet in transition states, distortions in this correlation may occur (17, 18). Hence, we may under- or overestimate biological effects based on our interpretation of the differential gene expression results 24 h after injection. Also, posttranscriptional (87) and posttranslational mechanisms (88) may account, among other things, for a non-linear correlation between mRNA and protein abundance. These shortcomings may also contribute to an explanation as to why the CLH, which features many of the same differentially regulated gene transcripts as the ILH, does not feature morphological alterations.

A previous study on gene expression revealed a mainly stage-specific (acute, latent, or chronic) gene expression profile in epileptogenesis (89). As such, the comparison of our gene set, representing relatively acute changes of kainic acid-induced status epilepticus, to previous data from a chronic time point of epileptogenesis (43) should be interpreted with caution.

Regarding the only marginal overlap of differential DNA methylation between the CLH and ILH and the non-existent overlap of genomic loci with the association between differential gene expression and differential DNA methylation, one possible cause is that the method for detecting differential DNA methylation, RRBS, does not include all CpGs (37). RRBS covers most CpGs in promoters and CpG islands (but not all) and has a low coverage at, for example, CpG shores and enhancers (37). We might *ergo* have missed specific genomic loci at which differential DNA methylation and differential gene expression coincide.

## Conclusion

In this study we found substantial changes in gene expression and DNA methylation 24 h after status epilepticus in the mouse hippocampus contralateral to the site of kainate injection. This begs the question why the CLH, in contrast to the ILH, does not develop hippocampal sclerosis? In the CLH we found an overweight of upregulated genes with potential anti-epileptogenic properties. Further, we detected a significantly lower number of differentially regulated genes in glia. We therefore hypothesize that both an overweight of upregulated genes and pathways with potential downstream anti-epileptogenic effects and a lower number of genes and pathways with pro-epileptogenic qualities in glia contribute to prevent epileptogenesis in the CLH. Gene expression changes in terms of nuclear mRNA may, however, only be one among many factors when it comes to finally determining cellular responses upon external stimuli. Also the role of DNA methylation for gene expression remains still uncertain in this model as we only found a marginal overlap of differentially methylated sites between the CLH and ILH. In order to further disentangle the cell- and stage-specific orchestration of epileptogenesis, it is essential to perform longitudinal animal studies including the investigation of acute and chronic time points of epileptogenesis. Finally, studies exploring neuronal and glial gene expression in human tissue are required in order to evaluate the clinical relevance of these findings.

## Data Availability Statement

Raw data is available under GEO accession code GSE153976.

## Ethics Statement

The animal study was reviewed and approved by Norwegian Food Safety Authority, the Center for Comparatice Medicine, Oslo University Hospital and the University of Oslo.

## Author Contributions

TB: conceptualization, data curation, formal analysis, investigation, methodology, project administration, validation, visualization, writing–original draft, and writing–review & editing. KH: conceptualization, funding acquisition, investigation, methodology, project administration, resources, supervision, validation, writing–original draft, and writing–review & editing. KS: conceptualization, funding acquisition, methodology, project administration, resources, supervision, and writing–review and editing. ET: conceptualization, methodology, project administration, resources, supervision, and writing–review & editing. CN: investigation and writing–review & editing. HH: conceptualization, investigation, methodology, project administration, writing–original draft, and writing–review & editing. MV: conceptualization, data curation, formal analysis, methodology, project administration, software, supervision, validation, visualization, writing–original draft, and writing–review & editing. All authors contributed to the article and approved the submitted version.

## Conflict of Interest

The authors declare that the research was conducted in the absence of any commercial or financial relationships that could be construed as a potential conflict of interest.

## Abbreviations

*CLH*, contralateral hippocampus comparison (specifically: comparison of the contralateral hippocampi of the kainic acid group *vs*. the sham group)
*GO*: Gene Ontology
*ILH*, ipsilateral hippocampus comparison (specifically: comparison of the ipsilateral hippocampi of the kainic acid group *vs*. the sham group)
*KEGG*: Kyoto Encyclopedia of Genes and Genomes
*mRNAseq*: mRNA sequencing
*mTLE-HS*: mesial temporal lobe epilepsy with hippocampal sclerosis
*RRBS*: reduced representation bisulfite sequencing.
